# Assessment of left atrial function by two-dimensional speckle tracking echocardiography in patients with metabolic-associated fatty liver disease

**DOI:** 10.1186/s43044-024-00462-w

**Published:** 2024-03-30

**Authors:** Sara I. El Sharkawy, Yousra Aboulenien, Basma Elnagar, Walaa Elkhalawany, Rehab Badawi

**Affiliations:** 1 Tanta Cardiology Department, Tanta University Faculty of Medicine, Tanta, Egypt; 2 Tropical Medicine and Infectious Diseases Department, Tanta University Faculty of Medicine, Tanta, Egypt

**Keywords:** Metabolic-associated fatty liver disease, Transient elastography, Transthoracic echocardiography

## Abstract

**Background:**

Metabolic-associated fatty liver disease (MAFLD) and cardiovascular diseases have mutual risk factors that contribute to pathogenic processes, increasing mortality and morbidity. This study aimed to evaluate variations in left ventricular (LV) structure and diastolic function among different subtypes and severity degrees of MAFLD patients, allowing early identification, intervention, and prevention of severe cardiac outcomes in high-risk populations.

**Results:**

The cross-sectional study included 142 MAFLD patients and 142 non-MAFLD participants as a control group. All participants underwent abdominal ultrasound, transient elastography, transthoracic echocardiography, tissue Doppler, and strain imaging. The results showed a significant impairment in the diastolic left ventricular function, as assessed with tissue Doppler, and the left atrial (LA) function, as evaluated with strain imaging, in the MAFLD group. Additionally, the left atrial stiffness was significantly higher in the MAFLD group.

**Conclusion:**

The use of strain imaging facilitated the detection of subtle impairments of the left atrial reservoir, contraction, conduit function, and left ventricular diastolic function in MAFLD patients.

## Background

The prevalence of non-alcoholic fatty liver disease (NAFLD) has been significantly rising in recent years and is currently estimated to affect a quarter of the population worldwide [1]. This disease encompasses a spectrum of conditions, ranging from simple steatosis to non-alcoholic steatohepatitis, which can further complicate into different grades of hepatic fibrosis, cirrhosis, or hepatocellular carcinoma [2, 3].

Eslam et al. recently suggested metabolic-associated fatty liver disease (MAFLD) as a more descriptive name for this spectrum of conditions than NAFLD [4].

Metabolic dysfunction results in MAFLD through increased fat deposition in the liver [5]. As MAFLD is in close association with metabolic disorders, patients with fatty liver are more susceptible to extrahepatic complications, such as cardiovascular diseases (CVDs) [6, 7]. CVDs and MAFLD have common risk factors, including diabetes, abnormal lipid metabolism, hypertension, insulin resistance, and inflammation [8, 9]. Furthermore, MAFLD has been identified as an independent risk factor for CVDs [10].

Symptoms of heart failure or other potentially fatal conditions may not manifest until the advanced stages of the illness, when MAFLD has already caused significant abnormalities in left ventricular (LV) diastolic function and cardiac structure [11–13].

As CVDs stand as one of the leading causes of global mortality and morbidity, accounting for nearly a third of all deaths and approximately half of the deaths from non-communicable diseases [14–16], recent research has focused on the effects of fatty liver accumulation on heart structure and function. Researchers aimed to discern an association between fatty liver subtypes, severity, and the risk of CVDs [11–13]. However, no clear link has been identified between MAFLD, diastolic function, and structural abnormalities of the LV.

The left atrium (LA) is crucial in diastolic LV filling and stroke volume, contributing to the various stages of the cardiac cycle. A noninvasive evaluation of LA function can be performed with ease and accessibility using transthoracic echocardiography [17].

In this study, we used Doppler echocardiography to evaluate the variations in diastolic function among different subtypes and severity degrees of MAFLD patients to identify early intervention and actively monitor high-risk population groups to prevent severe heart damage. Additionally, we utilized strain measured by 2D-STE (two-dimensional speckle tracking echocardiography) due to its recently proven ability to directly assess myocardial LA function and deformation, independent of the angle [18].

## Methods

In this cross-sectional study, we recruited patients with MAFLD from the Tropical and Cardiovascular Medicine departments of Tanta Faculty of Medicine in Egypt between March 2022 and December 2022. We enrolled 142 patients with liver steatosis, initially identified through liver ultrasound by the characteristic bright liver. Then, these patients were diagnosed with liver steatosis exceeding 237 dB/min using a controlled attenuation parameter (CAP). Additionally, 142 controls (without MAFLD) were included, attending the Cardiovascular Medicine Department for elective echocardiography after exclusion criteria were applied in both groups.

The Ethical Committee approved the study following the Helsinki Declaration (approval number: 35272\2\22) before its commencement. Each patient received comprehensive information about the study, had the opportunity to ask questions, and provided informed written consent for all necessary laboratory investigations and noninvasive scans (including abdominal and pelvic ultrasound, transient elastography, and echocardiography) before enrolling in the study. Furthermore, participants consented to publishing their results with any patient's identification.

All authors reviewed and approved the study's final content, and all authors had full access to the underlying data.

### Inclusion criteria

Male and female patients aged 18 or older who met the MAFLD diagnosis criteria were included. MAFLD was diagnosed when hepatic steatosis was present, along with at least one of the following three criteria:

1. Overweight/obesity (BMI 25 kg/m^2^).
2. Presence of type 2 diabetes mellitus.
3. Evidence of metabolic dysregulation, defined by the presence of at least two of the seven metabolic at-risk factors, which include the following: waist circumference in men/women ≥ 94/80 cm, blood pressure more than 130/85 mmHg, plasma triglycerides exceeding 150 mg/dL, HDL-cholesterol less than 40/50 mg/dL for men/women, prediabetes, HOMA-insulin resistance score 2.5, and plasma hs-CRP > 2 mg/dl [1].

### Exclusion criteria

- Aged < 18 years
- Alcohol consumption
- Chronic liver disease due to drug administration
- Autoimmune hepatitis
- Unwillingness to participate in the study
- History of ischemic heart disease, cardiomyopathy, or valvular heart disease
- Peripheral artery disease
- Implanted cardiac pacemaker
- History of myocardial infarction and stroke
- History of receiving drugs that cause steatosis (e.g., amiodarone)

### Methodology

A total of 284 participants were included, divided into two groups based on the presence of MAFLD. Group 1 consisted of 142 patients with MAFLD, while Group 2 consisted of 142 participants without MAFLD (controls). All patients underwent the following assessments:

1. Full history-taking
2. Clinical examination, including body mass index calculation and waist circumference measurement
3. Laboratory investigation, including liver functions, blood urea, serum creatinine, lipid profiles, and a complete blood picture
4. Abdominal imaging studies:
  I. Ultrasound on abdomen and pelvis: to evaluate liver condition, splenic size, and presence of ascites.
  II. Transient elastography (fibro-scan): The liver steatosis was identified using CAP, and the hepatic stiffness measurements were obtained by well-trained experts following the company's instructions using the 502 M and XL fibro-scan (echo Sens-France) probe. The scan was conducted with the patient in the supine position, and the right arm was wholly abducted from the intercostal transthoracic window on the right hepatic lobe.

We adopted the following CAP cutoff values, as used in another study, to indicate liver steatosis (S): S0 denoted no steatosis (237 dB/m), S1 for mild steatosis (ranging from 237.0 to 259.0 dB/m), S2 for moderate steatosis (ranging from 259.0 to 291.0 dB/m), and S3 for severe steatosis (ranging from 291.0 to 400.0 dB/m) [18]. The fibrosis cutoff values (F) were defined as follows: F0 for no fibrosis (< 5.5 kPa), F1 for mild fibrosis (ranging from 5.5 to 8.0 kPa), F2 for moderate fibrosis (ranging from 8.0 to 10.0 kPa), F3 for severe fibrosis (ranging from 11.0 to 16.0 kPa), and F4 for cirrhosis (> 16.0 kPa) [18].

5. Echocardiography examination

All echocardiographic acquisitions were conducted using the Vivid E9 ultrasound system (GE Vingmed Ultrasound, Horten, Norway) equipped with an M5S phased array transducer (2.5–5.0 MHz), following the guidelines of the American Society of Echocardiography [16]. The acquired data were transferred to an echo pack for offline analysis. Left ventricle end-systolic and end-diastolic volumes and ejection fraction (EF) were estimated using Simpson's modified biplane method. The assessment of LV and diastolic function involved pulse Doppler (mitral *E* wave, mitral *A* wave, and *E*/*A* ratio) and tissue Doppler imaging (early diastolic (*E*′), late diastolic velocity (*A*′), and the ratio of *E*/*E*′).

**Fig. 1:**
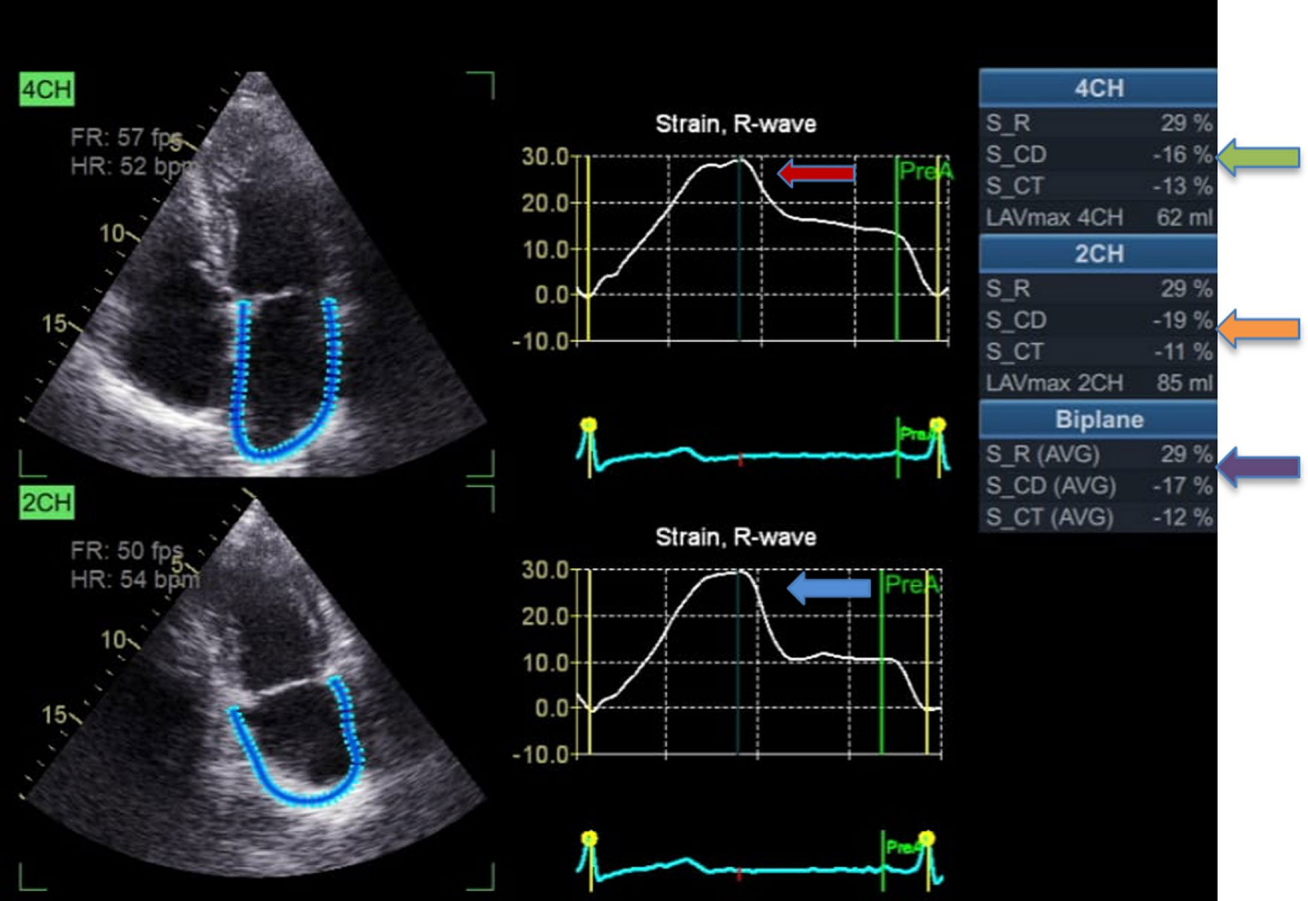
Measures of LA strain by speckle tracking. Red and blue arrows denote peak reservoir strains for the 4- and 2-chamber echocardiographic views, respectively. Green and orange arrows represent the automatic results for the reservoir, conduit, and contraction measures in the 4- and 2-chamber views, respectively. The purple arrow highlights the mean values for the two views

I. LA analysis: The left atrium focused on four chambers and two-chamber views to avoid LA foreshortening. The LA analysis utilized automated function imaging (AFI) software (GE Vingmed Ultrasound AS, Horten, Norway) explicitly dedicated to LA assessment. By placing two landmarks, one at the mitral annulus and the other at the atrial roof, the software traced the endocardium and defined the region of interest (ROI). The zero strain was set at the R-wave of the ECG (left ventricular end-diastole). The AFI software provided the left atrium strain values, including LA strain at reservoir phase (LASr), LA strain at conduit phase (LAScd), and LA strain at contractile phase (LASct) for each view, as well as their average. Additionally, it calculated the LA emptying fraction (LAEF), minimum (LA Vmin), maximum (LA Vmax), and pre-atrial contraction (LA V Pre-A) volumes for each single plane and biplane. LA Vmax was indexed to body surface area (BSA), expressed as LAVI max = LA Vmax /BSA. The LA stiffness index (LASI) was determined using the equation (*E*/*e*′/LAS-S).
II. Epicardial fat thickness was measured on the right ventricular free wall in at least two locations from parasternal longitudinal and transverse parasternal views.
III. Assessment of carotid atherosclerosis by intima-media wall thickness (IMT): Using a B mode with a 7.5–10 MHz linear phased array transducer with the patients lying supine and their neck extended and turned away from the examined side, common carotid arteries were examined using a posterior approach with both transverse and longitudinal scans (Fig. 1).

### Statistical analysis

The data were analyzed using the Statistical Package for Social Sciences (IBM SPSS Statistics), version 26 for Windows (IBM Corp., Armonk, NY, USA). Categorical variables (e.g., gender and smoking) were summarized as frequencies. The association between the categorical variables and the studied groups was tested using Pearson's Chi-Square test for independence. The Shapiro–Wilk test determined their distribution for the continuous numerical variables (e.g., age, ejection fraction). Following a normal distribution, numerical variables were summarized as the mean and standard deviation (SD). Comparisons were made using the independent-samples T-test (for two groups) or the one-way analysis of variance (ANOVA) test for three groups. Tukey's or Games–Howell post hoc tests were applied if the *p* value was significant (the choice of post hoc test was based on the homogeneity of variance test). Numerical variables not adhering to the normal distribution were summarized as the median and interquartile range (IQR, expressed as 25th–75th percentiles). Comparisons utilized the Mann–Whitney test for two groups or the Kruskal–Wallis test for three groups, followed by the Dunn–Bonferroni post hoc test if the *p* value was significant.

Spearman's rank-order correlation was performed between the numerical and/or ordinal factors. The correlation was categorized as weak (*r*_s_ < 0.3), moderate (*r*_s_ = 0.3–0.7), and strong (*r*_s_ > 0.7), regardless of the sign of the correlation coefficient.

Receiver operating characteristics (ROC) curve analysis identified the optimal cut-off points of the studied measurements, their sensitivity, specificity, and positive and negative predictive values (PPV and NPV). The area under the curve (AUC) was classified as excellent (= 0.9–1.0), good (= 0.8–0.9), fair (= 0.7–0.8), and poor (= 0.6–0.7).

Binomial logistic regression analysis was performed to assess the potential risk factors affecting the development of diastolic dysfunction in MAFLD patients. Factors with a *p* value < 0.1 in the univariate analysis were entered into the multivariate model. A *p* value < 0.05 indicates statistical significance for all tests.

## Results

In this cross-sectional study, a total of 284 participants were included. The participants were divided into two groups according to the presence of MAFLD: Group 1 consisted of 142 patients with MAFLD, and Group 2 consisted of 142 participants without MAFLD (controls). Regarding the baseline characteristics, the median age of both groups was 45 years. Group 1 had 36 (25.4%) males and 106 (74.6%) females, while Group 2 included 49 (34.5%) males and 93 (65.5%) females. A statistically significant increase was observed in the incidence of obesity, hypertension, diabetes mellitus, and hyperlipidemia in group I (*p* value < 0.001). The two groups showed no significant difference in liver enzymes (see Tables 1, 2).

**Table 1:** Demographic and clinical data of the studied groups

		Controls (*n* = 142)	Patients (*n* = 142)	*p* value
Age (years)	Median [IQR] (Range)	45.00 [40.00–49.00] (34.00–60.00)	45.00 [40.00–47.00] (34.00–56.00)	0.117 *Z*
Sex n (%)	Female	93 (65.5%)	106 (74.6%)	0.092 *X*^2^
	Male	49 (34.5%)	36 (25.4%)	
Smoker n (%)	No	90 (63.4%)	105 (73.9%)	0.055 *X*^2^
	Yes	52 (36.6%)	37 (26.1%)	
DM	No	114 (80.3%)	69 (48.6%)	< 0.001* *X*^2^
	Yes	28 (19.7%)	73 (51.4%)	
Weight (kg)	Mean ± SD	80.90 ± 5.15	91.74 ± 8.66	< 0.001* *t*
Height (m)	Mean ± SD	164.72 ± 6.67	163.72 ± 7.19	0.213 *t*
BSA	Mean ± SD	1.88 ± 0.09	1.95 ± 0.10	< 0.001* *t*
BMI (kg/m^2^)	Mean ± SD	29.77 ± 2.39	30.30 ± 3.32	0.111 *t*
Waist circumference (cm)	Mean ± SD	81.86 ± 6.19	97.26 ± 9.48	< 0.001* *t*
Systolic blood pressure (mmHg)	Mean ± SD	118.10 ± 5.11	140.40 ± 12.65	< 0.001* *t*
Diastolic blood pressure (mmHg)	Mean ± SD	75.50 ± 4.84	88.78 ± 6.79	< 0.001* *t*
Heart rate (beat/min)	Mean ± SD	86.82 ± 5.64	90.22 ± 7.35	< 0.001* *t*

IQR, Interquartile range (25th–75th percentiles); Max, Maximum; Min, Minimum; *n*, Number; SD, Standard deviation; *t*, Independent samples *T*-test; *X*^2^, Pearson’s Chi-square test of association; *Z*, Mann–Whitney test; BSA, Body surface area; BMI, body mass index

*Significant at *p* < 0.05

**Table 2:** Laboratory data of the studied groups

		Controls (*n* = 142)	Patients (*n* = 142)	*p* value
FBS	Mean ± SD	84.46 ± 8.37	106.96 ± 12.40	< 0.001* t
HbA1c %	Mean ± SD	5.08 ± 0.34	6.94 ± 1.57	< 0.001* t
SGPT	Median [IQR] (Range)	28.00 [25.00–30.00] (16.00–40.00)	25.50 [22.00–30.00] (16.00–66.00)	0.001* Z
SGOT	Median [IQR] (Range)	30.00 [28.00–35.00] (20.00–40.00)	27.00 [23.00–32.00] (16.00–54.00)	< 0.001* Z
Total cholesterol (mg/dL)	Mean ± SD	143.60 ± 9.88	220.30 ± 21.22	< 0.001* t
TG (mg/dL)	Mean ± SD	121.54 ± 26.88	161.96 ± 29.22	< 0.001* t
LDL (mg/dL)	Mean ± SD	53.40 ± 13.06	110.51 ± 23.15	< 0.001* t
HDL (mg/dL)	Mean ± SD	50.40 ± 7.83	40.60 ± 8.10	< 0.001* t

IQR, Interquartile range (25th–75th percentiles); Max, Maximum; Min, Minimum; *n*, Number; SD, Standard deviation; *t*, Independent samples *T*-test; *Z*, Mann–Whitney test; FBS, Fasting blood sugar

*Significant at *p* < 0.05

### Fibro-scan results

In MAFLD patients (Group 1), 41 patients had fibrosis grade 0 (52.8%), 56 patients had fibrosis grade I (39.4%), six patients had fibrosis grade II (4.3%), and five patients had fibrosis grade III (3.5%). In contrast, in the control group (Group 2), 128 patients had fibrosis grade 0 (90.1%), 11 patients had fibrosis grade I (7.8%), three patients had fibrosis grade II (2.1%), and no patients had fibrosis grade III, as illustrated in Table 3.

**Table 3:** Fibro-scan results of the studied groups

		Control group (*n* = 142)	MAFLD group (*n* = 142)	MAFLD nondiabetic subgroup (*n* = 69)	MAFLD diabetic subgroup (*n* = 73)	*p* value
Fibrosis grade *n* (%)	F0	128 (90.1%)	72 (52.8%)	41 (59.4%)	34 (46.6%)	< 0.001* *X*^2^
	F1	11 (7.7%)	56 (39.4%)	28 (40.6%)	28 (38.4%)	
	F2	3 (2.1%)	6 (4.3%)	0 (0.0%)	6 (8.2%)	
	F3	0 (0.0%)	5 (3.5%)	0 (0.0%)	5 (6.8%)	
Steatosis grade *n* (%)	S0	142 (100.0%)	0 (0.0%)	12 (17.4%)	2 (2.7%)	< 0.001* *X*^*2*^
	S1	0 (0.0%)	25 (17.6%)	16 (23.2%)	9 (12.3%)	
	S2	0 (0.0%)	42 (29.6%)	18 (26.1%)	17 (23.3%)	
	S3	0 (0.0%)	75 (52.8%)	23 (33.3%)	45 (61.6%)	
Fibrosis score (kb)	Median [IQR] (Range)	5.00 b,c [4.50–5.50] (3.50–8.00)	6.00 [5.00–7.00] (3.5–12)	5.50 a [5.00–7.00] (4.50–8.00)	6.00 a [5.00—7.00] (3.50—12.00)	< 0.001* *Z*
Steatosis score (dm)	Median [IQR] (Range)	110.50 b,c [99.00–123.00] (80.00–145.00)	285.00 [260.00–310.00] (100.00–399.00)	270.00 a,c [255.00–300.00] (100.00–366.00)	300.00 a,b [275.00–320.00] (150.00–399.00)	< 0.001* *Z*

IQR, Interquartile range (25th–75th percentiles); Max, Maximum; Min, Minimum; *n*, Number; SD, Standard deviation; *X*^2^, Pearson’s Chi-square test of association; Z, Kruskal–Wallis test

*Significant at *p* < 0.05; a, Significant difference from control group from post hoc test; b, Significant difference from nondiabetic group from post hoc test; c, Significant difference from diabetic group from post hoc test

The median fibrosis score in the MAFLD group was 6 kb, while the median fibrosis score in the control group was 5 kb. The median fibrosis score in the nondiabetic subgroup of MAFLD patients was 5.5 kb, whereas the diabetic subgroup demonstrated a score of 6.00 kb. Refer to Table 3.

The median steatosis score in the MAFLD group was 285 dm, while the control group exhibited a score of 220 dm. The median steatosis group in the nondiabetic subgroup of MAFLD patients was 270 dm, while in the diabetic subgroup, it was 300 dm, as presented in Table 3.

In post hoc analysis, both the difference in the median fibrosis score and median steatosis score were found to be significant when comparing the diabetic subgroup and nondiabetic subgroup of the MAFLD group separately to the control group, as illustrated in Table 3.

### Conventional echocardiographic findings

Regarding the echocardiographic characteristics of the participants, there were no significant differences between the two groups in terms of the LA diameter or the conventional parameters of LV structure and systolic function (LVEDD, LVESD, LVEF, and LVFS), as summarized in Table 4.

**Table 4:** Echocardiographic findings of studied groups

		Controls (*n* = 142)	Nondiabetic patients (*n* = 69)	Diabetic patients (*n* = 73)	*p* value
Carotid IMT (cm)	Mean ± SD	0.52 ± 0.18 b,c	0.67 ± 0.25 a	0.68 ± 0.27 a	< 0.001* *F*
LV EF%	Mean ± SD	62.70 ± 4.50	61.21 ± 3.79	62.38 ± 5.69	0.084 *F*
LV FS %	Mean ± SD	32.66 ± 3.80	31.79 ± 3.76	32.42 ± 5.46	0.279 *F*
E (m/s)	Mean ± SD	0.89 ± 0.12 b,c	0.76 ± 0.14 a	0.78 ± 0.17 a	< 0.001* *F*
LVEDD (mm)	Mean ± SD	48.00 ± 1.78	48.12 ± 2.40	48.35 ± 2.12	0.470 *F*
LVESD (mm)	Mean ± SD	28.92 ± 1.36	28.35 ± 1.90	28.75 ± 2.60	0.081 *F*
A (m/s)	Mean ± SD	0.83 ± 0.15 c	0.87 ± 0.14	0.90 ± 0.18 a	0.010* *F*
E/A	Mean ± SD	1.10 ± 0.19 b,c	0.98 ± 0.18 a	0.96 ± 0.22 a	< 0.001* *F*
e (m/s)	Mean ± SD	0.10 ± 0.01 b,c	0.08 ± 0.01 a	0.08 ± 0.02 a	< 0.001* *F*
E/e	Mean ± SD	8.64 ± 1.44 b,c	9.33 ± 2.07 a	9.54 ± 1.61 a	< 0.001* *F*
A	Median [IQR] (Range)	0.09 [0.08–0.10] (0.06–0.15)	0.09 [0.08–0.11] (0.06–1.20)	0.09 [0.08–0.14] (0.05–1.13)	0.135 *Z*
IVRT (msec)	Mean ± SD	64.68 ± 8.35 b,c	72.71 ± 11.81 a	73.88 ± 11.48 a	< 0.001* *F*
Lt atrial dimension (cm)	Mean ± SD	3.13 ± 0.10	3.12 ± 0.15	3.10 ± 0.15	0.246 *F*
LAV max (ml)	Mean ± SD	51.14 ± 5.27 b,c	53.92 ± 8.65 a	54.50 ± 9.27 a	0.015* *F*
LAVI	Mean ± SD	27.21 ± 2.61 b,c	28.74 ± 4.92 a	29.11 ± 5.05 a	0.010* *F*
LAV min (ml)	Mean ± SD	26.08 ± 4.53	25.42 ± 5.76	26.81 ± 7.25	0.426 *F*
LAV pre-A (ml)	Median [IQR] (Range)	37.50 [34.00–40.00] (27.00–51.00)	37.00 [34.00–42.50] (21.00–63.00)	39.50 [32.00–47.00] (29.00–76.00)	0.138 *Z*
LA EF (Avg) %	Median [IQR] (Range)	49.17 [45.00–54.55] (17.39–64.41)	50.50 [48.50–55.00] (40.00–62.00)	50.50 [47.00–54.00] (41.00–64.00)	0.124 *Z*
LA EV (Avg) (ml)	Median [IQR] (Range)	29.00 b,c [24.00–31.00] (8.00–38.00)	25.50 a [22.50–27.50] (17.00–42.00)	25.50 a [22.00–28.00] (16.00–34.00)	< 0.001* *Z*
Epicardial fat thickness (mm)	Mean ± SD	5.46 ± 2.07 b,c	8.75 ± 2.31 a	8.96 ± 2.34 a	< 0.001* *F*

*F*, One-way analysis of variance (ANOVA); IQR, Interquartile range (25th–75th percentiles); Max, Maximum; Min, Minimum; *n*, Number; SD, Standard deviation; *Z*, Kruskal–Wallis test; IMT, Intima-media thickness, LV EF, Left ventricular ejection fraction; LV FS, Left ventricular fractional shortening; LVEDD, Left ventricular end-diastolic dimension; LVESD, Left ventricular end-systolic dimension; IVRT, Isovolumetric relaxation time; LAV; Left atrial volume; LAVI, Left atrial volume index; LA EF, Left atrial emptying fraction; LA EV, Left atrial emptying volume

*Significant at *p* < 0.05; a, Significant difference from control group from post hoc test; b, Significant difference from nondiabetic group from post hoc test; c, Significant difference from diabetic group from post hoc test

However, Group 1 exhibited significantly higher left atrial volumes compared to Group 2, particularly in maximum left atrial volume (LAV max ml) (*p* = 0.015) and left atrial volume index (LAVI) in group 1 (*p* = 0.01), as presented in Table 4.

Furthermore, the results showed significant differences between the two groups' diastolic left ventricular function parameters. In MAFLD patients, there was a significant decrease in the peak velocity of the mitral E wave and E/A ratio (*p* < 0.001), accompanied by a significantly higher peak velocity of the mitral A wave and E/e ratio (*p* = 0.010). Post hoc analysis revealed that this difference in the diastolic LV function parameters was significant when comparing the diabetic and nondiabetic groups in the MAFLD groups to the respective control groups separately. Refer to Table 4.

Regarding pericardial fat thickness, it significantly increased in patients from Group 1 (*p* < 0.001). In post hoc analysis, the difference in pericardial fat thickness was significant when the diabetic and nondiabetic groups in the MAFLD groups were compared to the control groups separately (see Table 4).

Table 4 shows a significant increase in carotid intima thickness in the diabetic and nondiabetic subgroups of MAFLD patients compared to the control group (*p* < 0.001).

### LA strain measured by 2D-STE

When LA strain was used, significant impairment was observed in LA reservoir function (LA S-R average) function (*p* < 0.001), left atrial contraction function (LA S-CT average) function (*p* < 0.001), and LA conduit (LA S-CD average) function (*p* < 0.001) in Group 1 compared to Group 2 (*p* < 0.001). Additionally, Table 5 shows a significant decrease in the global longitudinal strain of left ventricular function (LV GLS) in Group 1 compared to Group 2 (*p* < 0.001).

**Table 5:** Results of LA and LV strain by 2D-STE in the studied groups

		Controls (*n* = 142)	Nondiabetic patients (*n* = 69)	Diabetic patients (*n* = 73)	*p* value
LA strain S-R(AVG)	Mean ± SD	30.88 ± 3.05 b,c	27.21 ± 3.56 a,c	25.42 ± 3.44 a,b	< 0.001* *F*
LA strain S-CD (AVG)	Median [IQR] (Range)	− 17.00 b,c [− 20.00 to − 16.00] (− 24.00 to − 11.00)	− 14.00 a [− 16.50 to − 13.00] (− 24.00 to − 11.00)	− 14.00 a [− 15.00 to − 13.00] (− 18.00 to − 8.00)	< 0.001* *Z*
LA S-CT(AVG)	Median [IQR] (Range)	− 13.00 b,c [− 14.00 to − 12.00] (− 18.00 to − 11.00)	− 11.00 a [− 13.5 to − 10.50] (− 14.00 to − 14.50)	− 11.00 a [− 13.00 to − 10.00] (− 14.00 to 15.00)	< 0.001* *Z*
LA stiffness index	Median [IQR] (Range)	0.29 b,c [0.24 to 0.31] (0.16 to 0.37)	0.33 a [0.28 to 0.41] (0.19 to 0.51)	0.38 a [0.31 to 0.43] (0.25 to 0.61)	< 0.001* *Z*
LV GLS	Median [IQR] (Range)	− 18.75 b,c [− 19.70 to − 18.00] (− 21.20 to − 17.30)	− 18.15 a [− 19.55 to − 17.50] (− 21.00 to − 14.10)	− 18.00 a [− 19.00 to − 17.10] (− 26.00 to − 14.80)	< 0.001* *Z*
LA subclinical diastolic dysfunction	Absent	142 (100.0%)	66 (95.7%)	56 (76.7%)	0.019* *X*^2^ #
	Present	0 (0.0%)	3 (4.3%)	17 (23.3%)	

*F*, One-way analysis of variance (ANOVA); IQR, Interquartile range (25th–75th percentiles); Max, Maximum; Min, Minimum; *n*, Number; SD, Standard deviation; *X*^*2*^, Pearson’s Chi-square test of association; *Z*, Kruskal–Wallis test; S-R, Strain reservoir, S-CD, Strain conduit, S-CT, Strain contractile, LV GLS, Left ventricular global longitudinal strain, AVG, Average

*Significant at *p* < 0.05; a, Significant difference from control group from post hoc test; b, Significant difference from nondiabetic group from post hoc test; c, Significant difference from diabetic group from post hoc test. # The association was assessed between nondiabetic and diabetic MAFLD patients only

Additionally, LA stiffness was significantly higher in MAFLD patients than in controls (*p* < 0.001), as illustrated in Table 5.

In post hoc analysis, we found that the difference in LA S-R average, LA S-CD average, LA S-CT average, and LA stiffness were also significant when the diabetic and nondiabetic subgroups in the MAFLD groups were compared to the control groups separately. Refer to Table 5.

The prevalence of subclinical diastolic dysfunction within the 142 patients in the MAFLD group was 14.08% (95% confidence interval: 8.82 to 20.91%). In the MAFLD group, the prevalence of subclinical diastolic dysfunction within the 69 nondiabetic cases was 4.35% (95% confidence interval: 0.91 to 12.18%) while the prevalence within the 73 diabetic cases was 23.29% (95% confidence interval: 14.19 to 34.65%). Refer to Table 5 for the number and percentage of cases.

### Receiver operating characteristics analysis

**Fig. 2:**
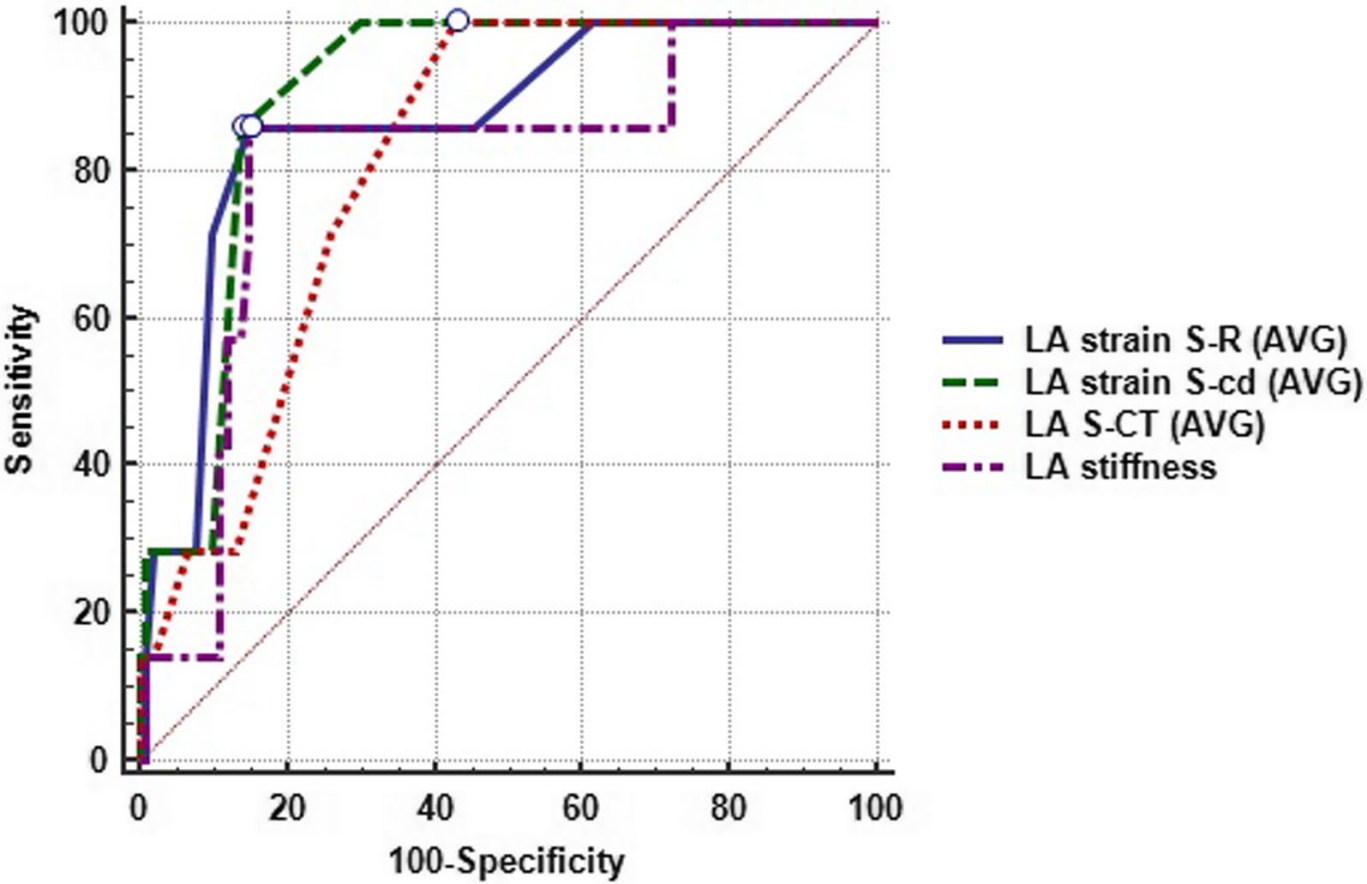
Receiver operating characteristics curve for assessing the diagnostic performance of studied measurements

The receiver operating characteristics (ROC) curve was analyzed to assess the diagnostic performance of the study's measurements for LA diastolic dysfunction. All measurements had an AUC above 0.8, indicating good discriminatory power. LA S-Cd (AVG) had the most significant AUC (AUC = 0.899), indicating the best discriminatory power, and pairwise comparisons showed a significant difference compared to LA S-CT(AVG) and LA stiffness (AUCs = 0.813 and 0.806, respectively). The second highest AUC was observed with LA strain S-R (AVG) (AUCs = 0.866), which was significantly higher than LA stiffness but not substantially different from LA S-CT (AVG). There was no significant difference between LA S-CD (AVG) and LA S-R (AVG), as shown in Table 6 and Fig. 2.

**Table 6:** LA strain and stiffness as predictors of subclinical LV diastolic dysfunction

	AUC	95% CI	p value#	Cut-off	Sens. (%)	Spec. (%)	PPV (%)	NPV (%)
LA strain S-R(AVG)	0.866 d	0.789 to 0.943	< 0.001*	≤ 25	85.7	85.0	30.0	98.7
LA strain S-CD (AVG)	0.899 c,d	0.859 to 0.940	< 0.001*	> − 14	85.7	86.0	31.6	98.8
LA S-CT (AVG)	0.813 b	0.750 to 0.875	< 0.001*	> − 13	100.00	57.0	14.9	100.0
LA stiffness Index	0.806 a,b	0.705 to 0.906	< 0.001*	> 0.39	85.71	84.95	30.0	98.7

AUC, Area under ROC curve; CI, Confidence interval; #, *p* value from a test comparing AUC to the null hypothesis AUC of 0.5; Sens, Sensitivity; Spec., Specificity; PPV, Positive predictive value; NPV, Negative predictive value; S-R, Strain reservoir; S-CD, Strain conduit; S-CT, Strain contractile; AVG, Average

*Significant at *p* < 0.05; a, Significant difference from LA strain S-R (AVG); b, Significant difference from LA strain S-CD (AVG); c, Significant difference from LA S-CT (AVG); d, Significant difference from LA stiffness

### Correlations

The steatosis score had a significant strong positive correlation with the severity of LV diastolic dysfunction (*r*_s_ = 0.760, *p* < 0.001) and had a moderate positive correlation with LA stiffness (*r*_s_ = 0.470, *p* < 0.001). Table 7 reveals that the steatosis score showed a stronger correlation with the severity of LV diastolic dysfunction and LA stiffness than the fibrosis score.

**Table 7:** Correlations between liver fibrosis and steatosis scores and various left atrial parameters

		Fibrosis score	Steatosis score	LA stiffness	Severity of LA diastolic dysfunction
Fibrosis score	*r*_s_	–	0.332	0.089	0.280
	*p* value	–	< 0.001*	0.125	< 0.001*
Steatosis score	*r*_s_	0.332	–	0.470	0.760
	*p* value	< 0.001*	–	< 0.001*	< 0.001*
LA strain S-R(AVG)	*r*_s_	− 0.247	− 0.492	− 0.524	− 0.611
	*p* value	< 0.001*	< 0.001*	< 0.001*	< 0.001*
LA strain S-CD (AVG)	*r*_s_	0.296	0.429	0.467	0.525
	*p* value	< 0.001*	< 0.001*	< 0.001*	< 0.001*
LA S-CT(AVG)	*r*_s_	0.107	0.264	0.287	0.374
	*p* value	0.064	< 0.001*	< 0.001*	< 0.001*
LA stiffness	*r*_s_	0.089	0.470	–	0.589
	*p* value	0.125	< 0.001*	–	< 0.001*

*r*_s_, Coefficient of Spearman rank-order correlation; S-R, Strain reservoir, S-CD, Strain conduit, S-CT, Strain contractile, AVG, Average

*Significant at *p* < 0.05

The steatosis score was also found to have a significant moderate negative correlation with the LS strain S-R average (*r*_s_ = − 0.492, *p* < 0.001), a significant moderate positive correlation with LA strain S-CD (*r*_s_ = 0.429, *p* < 0.001), and a significantly close-to-moderate positive correlation with LA strain S-CT (*r*_s_ = 0.264, *p* < 0.001). Additionally, the steatosis score showed a stronger correlation with LA strain function parameters than the fibrosis score (see Table 7).

The LA stiffness had a significant moderate negative correlation with LA S-R (*r*_s_ = − 0.524, *p* < 0.001), a significant moderate positive correlation with LA S-CD (*r*_s_ = 0.467, *p* < 0.001), and a significant close-to-moderate positive correlation with LA S-CT (*r*_s_ = 0.287, *p* < 0.001). Additionally, LA stiffness showed a significant moderate positive correlation with the severity of LV diastolic dysfunction (*r*_s_ = 0.589, *p* < 0.001), as shown in Table 7.

The LA S-R exhibited a significant moderate negative correlation with the severity of LV diastolic dysfunction (*r*_s_ = − 0.611, *p* < 0.001). At the same time, the LA S-CD showed a significant moderate positive correlation (*r*_s_ = 0.525, *p* < 0.001), and the LA S-CT demonstrated a significant moderate positive correlation as well with the severity of LV diastolic dysfunction (*r*_s_ = 0.374, *p* < 0.001) (see Table 7).

### Using univariate and multivariate regression analysis to determine risk factors for the development of diastolic dysfunction in MAFLD

In univariate analysis, diabetes, TC ≥ 200 mg/dL, and HDL < 40 mg/dL emerged as significant risk factors for the development of diastolic dysfunction in MAFLD patients (Table 8). However, in multivariate regression analysis, diabetes and HDL < 40 mg/dL were identified as independent risk factors for the development of diastolic dysfunction in MAFLD patients (Table 8).

**Table 8:** Univariate and multivariate analysis of risk factors for the development of diastolic dysfunction in MAFLD patients

	Univariate analysis		Multivariate analysis model	
	*p* value	Unadjusted OR (95% CI)	*p* value	Adjusted OR (95% CI)
Age (years)	0.755	0.987 (0.907–1.074)	–	–
Male sex	0.994	1.004 (0.372–2.706)	–	–
DM	< 0.001*	21.048 (5.956–74.376)	0.001*	13.505 (2.863–63.696)
BMI (kg/m^2^)	0.055	0.865 (0.745–1.003)	0.331	0.891 (0.706–1.125)
Waist circumference (cm)	0.776	0.993 (0.945–1.043)	–	–
Total cholesterol	0.049*	1.030 (1.000–1.062)	0.136	1.033 (0.990–1.078)
LDL (mg/dL)	0.113	1.019 (0.996–1.043)	–	–
HDL (mg/dL)	< 0.001*	1.167 (1.081–1.259)	0.001*	1.231 (1.089–1.391)
Triglycerides (mg/dL)	0.094	0.987 (0.972–1.002)	0.805	0.997 (0.970–1.024)
Liver fibrosis	0.097	1.589 (0.920–2.743)	0.729	0.843 (0.320–2.220)
Liver steatosis	0.836	1.049 (0.665–1.656)	–	–

CI, Confidence interval; OR, Odds ratio; BMI, Body mass index

*Significant at *p* < 0.05

## Discussion

Our study was designed to evaluate LA function in patients with metabolic-associated fatty liver disease using 2D-speckle tracking echocardiography based on strain image parameters.

Clinical studies initially used conventional and tissue Doppler echocardiography to assess cardiac dysfunction in individuals with metabolic syndrome (MetS). The downsides of these approaches such as poor sensitivity, angle dependence, and difficulty in detecting modest impairment in myocardial contractility when assessing cardiac function have been well-documented [19].

LA function is commonly evaluated using traditional 2D echocardiography-derived volumetric measurements [20]. However, 2D echocardiography is not as accurate as cardiovascular magnetic resonance (CMR) imaging, the gold standard in volumetric quantification, as it tends to underestimate LA volumes [21]. LA volumes and function can also be assessed using cardiac computed tomography [22]; however, it poses the risk of ionizing radiation exposure and is not routinely used in clinical practice. Therefore, our study used the LA strain assessment using 2D speckle tracking due to its ability to evaluate atrial deformity from any perspective, enabling the tracking of LA phasic function and facilitating the early detection of subclinical cardiac dysfunction, even in those with normal LA size [23]. Additionally, this technique can overcome the limitations of volumetric assessment, which essentially relies on geometric assumptions and loading conditions [24]. LA strain is essential in classifying the degree of LV diastolic dysfunction [25].

One of the essential pathophysiological components of heart failure with preserved LV ejection fraction (HFpEF) is left atrial dysfunction [26].

There is substantial evidence that MetS affects the structure and function of the left heart, thereby reducing LV diastolic compliance and consequently increasing LV filling pressures, negatively impacting LA function [26].

LA function contributes through three components in LV filling: the systolic reservoir phase, the diastolic conduit phase, and the late diastolic active contractile component in individuals with sinus rhythm [26].

To ensure early management of patients before structural remodeling of the heart, diagnosing the preclinical functional changes that usually precede these structural changes in the heart chambers early is advisable [11].

Indices of LV and LA dimensions and LV systolic function showed non-significant differences between the two groups (LVEDD, LVESD, LA Diameter, LVEF, and LVFS). This balance in the baseline parameters between the two groups allowed us to study the subtle changes in LA function in MAFLD patients and build on the results of the previous small studies conducted in this field, especially considering the high prevalence of metabolic syndrome in the Middle East [27].

The results revealed impaired left ventricular diastolic function assessed by tissue Doppler and early subclinical LV systolic dysfunction assessed with GLS in MAFLD patients. This finding aligns with some results published in the literature. Fotbolcu et al. [28] demonstrated, using tissue Doppler echocardiography, that individuals with MAFLD (diagnosed by ultrasound) exhibit slower early diastolic relaxation (e) velocities than control participants. Additionally, compared to healthy controls, global LV longitudinal strain and strain rate in systole were reduced in MAFLD patients [29]. However, their study included a small population (a quarter of the number in our study), making our result more reliable. Furthermore, they did not use strain imaging to assess the subtle changes in LA function. In their study, the MAFLD patients were nondiabetic. Although this has the advantage of excluding diabetes as a factor in causing diastolic dysfunction, it might have led to the exclusion of many patients as diabetes is prevalent among MAFLD patients. In our study, 51.4% of the MAFLD patients had diabetes, and 49.6% were not diabetic. The results revealed significant impairment in LV diastolic function assessed by tissue Doppler in the diabetic and nondiabetic subgroups compared to the control patients.

Additionally, the research on coronary artery risk development in young adults (CARDIA) found that patients with MAFLD exhibited poorer absolute global longitudinal strain, more significant LV filling pressure, and lower e' velocity than the control group [30]. They included a large population in their study. However, they used Doppler and tissue Doppler imaging markers to provide insight into the association between MAFLD and heart failure, especially to define diastolic dysfunction. In contrast to our research, using the LA strain has provided insight into diagnosing diastolic dysfunction early.

Additionally, the findings demonstrated a significant negative correlation between the severity of diastolic dysfunction and the mean LA reservoir function in MAFLD patients compared to controls (*p* value = 0.001). This aligns with other literature demonstrating a significant negative correlation between LA reservoir strain and LA fibrosis, associated with invasively measured LV filling pressure, a marker of diastolic dysfunction [31, 32]. However, one of these studies assessed this correlation in patients with mitral valve (MV) disease who underwent MV surgery, while the other studied patients with non-valvular atrial fibrillation (AF) [31, 32]. Both of these patient categories are known to be at high risk of LA remodeling and fibrosis, which can affect the reservoir function. Our study confirmed this correlation even in MAFLD patients (diabetic and nondiabetic) using the LA strain.

This finding was consistent with the results of Decoin et al., who observed a significant reduction in the LA reservoir function in the group with MAFLD and severe fibrosis compared to the non-MAFLD group [33].

Our results agreed with those of Decoin et al. [33], as they revealed that the MAFLD group had substantially lower mean LA conduit and contraction function than the controls (*p* < 0.001). The left atrial volume index (LAVI) was also considerably more remarkable in the MAFLD group (*p* = 0.010). Additionally, our results revealed significant differences in the previous parameters in both the diabetic and nondiabetic subgroups of MAFLD patients when compared to the control group.

Our explanation for the previous data is that the increase in LA fibrosis resulted in reduced LA compliance and a trend toward depressed contractility of the LA in MAFLD patients, which was reflected in an increased LA volume index.

Consistent with our results, Ning et al. found that the mean LA reservoir and conduit functions were significantly lower for the metabolic syndrome patients than the controls when they used strain/strain rate (SR) imaging to investigate the effect of MetS on LA function in a total of 177 MetS patients and 156 normal subjects who underwent echocardiography. Despite this, the average LA contraction function of their controls and MetS patients was not significantly different [11]

Our results suggested the following cutoff points: LA S-R ≤ 25, LA S-CD >  − 14, LA S-CT >  − 13, and LA stiffness > 0.39, which appear to be good indicators for excluding subclinical LV diastolic dysfunction. However, they have limited utility in predicting positive subclinical LV diastolic dysfunction. These cut-off points could be tested in future studies with larger populations.

A moderate positive correlation was found between LA stiffness and the severity of LV diastolic dysfunction. Additionally, the LA reservoir and conduit strain functions had a stronger correlation to LA stiffness and the severity of LV diastolic dysfunction than the LA strain contraction function.

Furthermore, our results revealed that the correlation between the steatosis score and LA function, assessed by either tissue Doppler or strain rate, is stronger than the correlation between the fibrosis score and LA function. This aspect may require further investigation in future research.

Regarding the epicardial fat thickness, our results were consistent with the results of the meta-analysis of thirteen case–control studies, which included 2260 patients, conducted by Liu et al., and the meta-analysis of non-randomized observational studies performed by Orci et al., which included 3610 patients. Compared to controls, both studies showed a significant increase in pericardial fat thickness in the MAFLD subjects compared to the controls, thereby establishing the association between MAFLD and pericardial fat thickness [34, 35]. Our results revealed that this significant difference is in both the diabetic and nondiabetic subgroups of MAFLD patients, allowing us to exclude diabetes as the sole cause of increased epicardial fat thickness.

Regarding the carotid intima-media thickness, Shao et al. found that higher values for this parameter are linked to higher levels of liver stiffness in individuals with MAFLD [36]. The present study revealed a significant difference in the carotid intima-media thickness between the diabetic and nondiabetic subgroups of MAFLD patients and the control group. Therefore, these findings could suggest that the cardiovascular risk assessment of these patients and appropriate therapies might be aided by screening and surveillance for early atherosclerosis.

## Limitations

1. A larger sample of patients is needed to get more reliable results.
2. The study population was drawn from a single center and may not represent the broader population.
3. We know that liver biopsies, not fibro-scans, are considered the gold standard for determining the severity of MAFLD. However, liver biopsies are invasive and carry the risk of pain and other problems.
4. Fibro-scan may provide less accurate results due to poor echogenicity, narrow rib spaces, or ascites in obese cases. Additionally, it may underestimate or misdiagnose the stage of liver fibrosis in patients with mild hepatic inflammation. However, we tried to overcome these limitations by allowing only experts and highly specialized doctors to perform the scan on the included patients.
5. Echocardiogram results depend on obtaining optimal views. Therefore, only expert cardiologists were allowed to perform the echocardiography scans on the study population.
6. MRI is a gold-standard technique to measure epicardial fat; however, transthoracic echo is more available and less costly.
7. Limitations of the study design as a cross-sectional study:
8. Determining whether the exposure or outcome came first is difficult, leading to potential reverse causality as cross-sectional studies measure prevalence rather than incident cases. The data will always reflect determinants of survival as well as etiology.
9. Inability to measure incidence.
10. The associations identified may be challenging to interpret.
11. Susceptible to biases such as responder bias, recall bias, interviewer bias, and social acceptability bias.
12. Although some of the correlations in our study were statistically significant, they might not be clinically significant due to the cross-sectional design of our study, making all the findings suggestive.

Therefore, a case–control study is recommended in future research to provide a better relationship between causality and effect.

## Conclusions

Our study suggested significant impairment of LV diastolic function by echocardiographic tissue Doppler parameters, substantial impairment of LA function by 2D strain imaging, a notable increase in pericardial fat thickness, and a significant increase in carotid intima-media thickness in both diabetic and nondiabetic subgroups of MAFLD patients when compared to the non-MAFLD group. The categorization of MAFLD patients based on diabetic status helped to confirm that diabetes was not the main factor causing the significant difference between the MAFLD and non-MAFLD groups in these parameters. Additionally, our study suggested that the liver steatosis score correlates better than the liver fibrosis score with LA stiffness, function, and severity of LV diastolic dysfunction.

## Data Availability

The datasets used and/or analyzed during the current study are available from the corresponding author on reasonable request.

## Abbreviations

2D-STE: Two-dimensional speckle tracking echocardiography
AFI: Automated function imaging
AUC: Area under the curve
BMI: Body mass index
BSA: Body surface area
CAP: Controlled attenuation parameter
CVDs: Cardiovascular diseases
ECG: Electrocardiogram
FBS: Fasting blood sugar
GLS: Global longitudinal strain
HDL: High-density lipoprotein
HFpEF: Heart failure with preserved ejection fraction
HOMA: Homeostatic model assessment
Hs CRP: High-sensitivity lipoprotein
IMT: Intima-media thickness
IQR: Interquartile range
IVRT: Isovolumic relaxation time
LA: Left atrium
LA Vmax: Left atrium maximum volume
LA Vmin: Left atrium minimum volume
LA V pre-A: Pre-atrial contraction volume
LAEF: Left atrium emptying fraction
LA S-CD: Left atrial strain at conduit phase
LA S-CT: Left atrial strain at contraction phase
LASI: Left atrium stiffness index
LA S-R: Left atrial strain at reservoir phase
LAVI: Left atrium volume index
LV: Left ventricle
MAFLD: Metabolic-associated fatty liver disease
Mets: Metabolic syndrome
MRI: Magnetic resonance imaging
NPV: Negative predictive value
PPV: Positive predictive value
ROC: Receiver operator characteristic
ROI: Region of interest
SD: Standard deviation

## References

[1] Lazarus JV, Mark HE, Anstee QM, Arab JP, Batterham RL, Castera L, Cortez-Pinto H, Crespo J, Cusi K, Dirac MA, Francque S (2022). Advancing the global public health agenda for NAFLD: a consensus statement. Nat Rev Gastroenterol Hepatol.

[2] Anstee QM, Targher G, Day CP (2013). Progression of NAFLD to diabetes mellitus, cardiovascular disease or cirrhosis. Nat Rev Gastroenterol Hepatol.

[3] McPherson S, Hardy T, Henderson E, Burt AD, Day CP, Anstee QM (2015). Evidence of NAFLD progression from steatosis to fibrosing-steatohepatitis using paired biopsies: implications for prognosis and clinical management. J Hepatol.

[4] Eslam M, Newsome PN, Sarin SK, Anstee QM, Targher G, Romero-Gomez M, Zelber-Sagi S, Wong VW, Dufour JF, Schattenberg JM, Kawaguchi T (2020). A new definition for metabolic dysfunction-associated fatty liver disease: an international expert consensus statement. J Hepatol.

[5] Gofton C, George J (2021). Updates in fatty liver disease: Pathophysiology, diagnosis and management. Aust J Gen Pract.

[6] Patel SS, Siddiqui MS (2019). Current and emerging therapies for non-alcoholic fatty liver disease. Drugs.

[7] Li J, Zou B, Yeo YH, Feng Y, Xie X, Lee DH, Fujii H, Wu Y, Kam LY, Ji F, Li X (2019). Prevalence, incidence, and outcome of non-alcoholic fatty liver disease in Asia, 1999–2019: a systematic review and meta-analysis. Lancet Gastroenterol Hepatol..

[8] Dongiovanni P, Paolini E, Corsini A, Sirtori CR, Ruscica M (2021). Non-alcoholic fatty liver disease or metabolic dysfunction-associated fatty liver disease diagnoses and cardiovascular diseases: From epidemiology to drug approaches. Eur J Clin Investig.

[9] Shao C, Wang J, Tian J, Tang YD (2020) Coronary artery disease: from mechanism to clinical practice. In: Wang M, editor. Coronary artery disease: therapeutics and drug discovery, advances in experimental medicine. Springer Nature Singapore Pte Ltd.; 2020. pp 1–36. 10.1007/978-981-15-2517-9_1 32246442

[10] Abdallah LR, de Matos RC, de Souza YP, Vieira-Soares D, Muller-Machado G, Pollo-Flores P (2020). Non-alcoholic fatty liver disease and its links with inflammation and atherosclerosis. Curr Atheroscler Rep.

[11] Parvanescu T, Vitel A, Sporea I, Mare R, Buz B, Bordejevic DA (2021). Significant association between left ventricular diastolic dysfunction, left atrial performance and liver stiffness in patients with metabolic syndromeand non-alcoholic fatty liver disease. Diabetes Metab Syndr Obes.

[12] Chung GE, Lee JH, Lee H, Kim MK, Yim JY, Choi SY, Kim YJ, Yoon JH, Kim D (2018). Non-alcoholic fatty liver disease and advanced fibrosis are associated with left ventricular diastolic dysfunction. Atherosclerosis.

[13] Makker J, Tariq H, Bella JN, Kumar K, Chime C, Patel H, Kamal MU, Shaikh D, Vootla V, Bajantri B, Gomceli U (2019). Preclinical cardiac disease in non-alcoholic fatty liver disease with and without metabolic syndrome. Am J Cardiovasc Dis.

[14] Wei J, Liu S, Wang X, Li B, Qiao L, Wang Y, Zhu M (2021) Efficacy and safety of shexiang baoxin pill for coronary heart disease after percutaneous coronary intervention: A systematic review and meta-analysis. Evid Based Complement Altern Med 2021 10.1155/2021/2672516 PMC8684521 34931125

[15] Roth GA, Mensah GA, Fuster V (2020). The global burden of cardiovascular diseases and risks: a compass for global action. J Am Coll Cardiol.

[16] Roth GA, Mensah GA, Johnson CO, Addolorato G, Ammirati E, Baddour LM (2020). Global burden of cardiovascular diseases and risk factors, 1990–2019: update from the GBD 2019 study. J Am Coll Cardiol.

[17] Roşca M, Lancellotti P, Popescu BA, Piérard LA (2011). Left atrial function: pathophysiology, echocardiographic assessment, and clinical applications. Heart.

[18] Kocabay G, Karabay CY, Colak Y, Oduncu V, Kalayci A, Akgun T, Guler A, Kirma C (2014). Left atrial deformation parameters in patients with non-alcoholic fatty liver disease: a 2D speckle tracking imaging study. Clin Sci.

[19] Sunderji I, Singh V, Fraser AG (2020). When does the E/e′ index not work? The pitfalls of oversimplifying diastolic function. Echocardiography.

[20] Lang RM, Badano LP, MorAvi V (2015). Recommendations for cardiac chamber quantification by echocardiography in adults: an update from the American society of echocardiography and the European association of cardiovascular imaging. J Am Soc Echocardiogr.

[21] Kühl JT, Lønborg J, Fuchs A (2012). Assessment of left atrial volume and function: a comparative study between echo-cardiography, magnetic resonance imaging and multi slice computed tomography. Int J Cardiovasc Imaging.

[22] Szilveszter B, Nagy AI, Vattay B (2020). Left ventricular and atrial strain imaging with cardiac computed tomography: validation against echocardiography. J Cardiovasc Comput Tomogr.

[23] Muranaka A, Yuda S, Tsuchihashi K (2009). Quantitative assessment of left ventricular and left atrial functions by strain rate imaging in diabetic patients with and without hypertension. Echocardiography.

[24] Genovese D, Singh A, Volpato V (2018). Load dependency of left atrial strain in normal subjects. J Am Soc Echocardiogr.

[25] Singh A, Addetia K, Maffessanti F, Mor-Avi V, Lang RM (2017). LA strain for categorization of lv diastolic dysfunction. JACC Cardiovasc Imaging.

[26] Singh A, Addetia K, Maffessanti F, Mor-Avi V, Lang RM (2017). LA strain for categorization of LV diastolic dysfunction. JACC Cardiovasc Imaging.

[27] Ansarimoghaddam A, Adineh HA, Zareban I, Iranpour S, HosseinZadeh A, Kh F (2018). Prevalence of metabolic syndrome in Middle-East countries: meta-analysis of cross-sectional studies. Diabetes Metab Syndr.

[28] Fotbolcu H, Yakar T, Duman D, Karaahmet T, Tigen K, Cevik C, Kurtoglu U, Dindar I (2010). Impairment of the left ventricular systolic and diastolic function in patients with non-alcoholic fatty liver disease. Cardiol J.

[29] Karabay CY, Kocabay G, Kalayci A, Colak Y, Oduncu V, Akgun T, Kalkan S, Guler A, Kirma C (2014). Impaired left ventricular mechanics in non-alcoholic fatty liver disease: a speckle-tracking echocardiography study. Eur J Gastroenterol Hepatol.

[30] VanWagner LB, Wilcox JE, Colangelo LA, Lloyd-Jones DM, Carr JJ, Lima JA, Lewis CE, Rinella ME, Shah SJ (2015). Association of non-alcoholic fatty liver disease with subclinical myocardial remodeling and dysfunction: a population-based study. Hepatology.

[31] Her AY, Choi EY, Shim CY, Song BW, Lee S, Ha JW, Rim SJ, Hwang KC, Chang BC, Chung N (2012). Prediction of left atrial fibrosis with speckle tracking echocardiography in mitral valve disease: a comparative study with histopathology. Korean Circ J.

[32] Pilichowska-Paszkiet E, Baran J, Sygitowicz G, Sikorska A, Stec S, Kułakowski P, Zaborska B (2018). Non-invasive assessment of left atrial fibrosis. Correlation between echocardiography, biomarkers, and electroanatomical mapping. Echocardiography.

[33] Decoin R, Butruille L, Defrancq T, Robert J, Destrait N, Coisne A, Aghezzaf S, Woitrain E, Gouda Z, Schino S, Klein C (2022). High liver fibrosis scores in metabolic dysfunction-associated fatty liver disease patients are associated with adverse atrial remodeling and atrial fibrillation recurrence following catheter ablation. Front Endocrinol.

[34] Liu B, Li Y, Li Y, Liu Y, Yan Y, Luo A, Ren H, She Q (2019). Association of epicardial adipose tissue with non-alcoholic fatty liver disease: a meta-analysis. Hep Intl.

[35] Orci LA, Jornayvaz FR, Toso C, Gariani K (2022). Systematic review and meta-analysis of the usefulness of epicardial fat thickness as a non-invasive marker of the presence and severity of non-alcoholic fatty liver disease. Biomedicines.

[36] Shao C, Ye J, Li F, Lin Y, Wu T, Wang W, Feng S, Zhong B (2020). Early predictors of cardiovascular disease risk in non-alcoholic fatty liver disease: non-obese versus obese patients. Dig Dis Sci.

